# Sharing best practices through online communities of practice: a case study

**DOI:** 10.1186/1478-4491-8-25

**Published:** 2010-11-12

**Authors:** Annamma Udaya Thomas, Grace P Fried, Peter Johnson, Barbara J Stilwell

**Affiliations:** 1 Jhpiego Corporation, 1615 Thames Street, Baltimore, MD 21212, USA; 2 The Johns Hopkins University, 3400 N. Charles Street, Baltimore, 21218, USA; 3 IntraHealth International, 6340 Quadrangle Drive, Chapel Hill, NC 27517, USA

## Abstract

**Introduction:**

The USAID-funded Capacity Project established the Global Alliance for Pre-Service Education (GAPS) to provide an online forum to discuss issues related to teaching and acquiring competence in family planning, with a focus on developing countries' health related training institutions. The success of the Global Alliance for Nursing and Midwifery's ongoing web-based community of practice (CoP) provided a strong example of the successful use of this medium to reach many participants in a range of settings.

**Case description:**

GAPS functioned as a moderated set of forums that were analyzed by a small group of experts in family planning and pre-service education from three organizations. The cost of the program included the effort provided by the moderators and the time to administer responses and conduct the analysis.

**Discussion and evaluation:**

Family planning is still considered a minor topic in health related training institutions. Rather than focusing solely on family planning competencies, GAPS members suggested a focus on several professional competencies (e.g. communication, leadership, cultural sensitivity, teamwork and problem solving) that would enhance the resulting health care graduate's ability to operate in a complex health environment. Resources to support competency-based education in the academic setting must be sufficient and appropriately distributed. Where clinical competencies are incorporated into pre-service education, responsible faculty and preceptors must be clinically proficient. The interdisciplinary GAPS memberships allowed for a comparison and contrast of competencies, opportunities, promising practices, documents, lessons learned and key teaching strategies.

**Conclusions:**

Online CoPs are a useful interface for connecting developing country experiences. From CoPs, we may uncover challenges and opportunities that are faced in the absorption of key public health competencies required for decreasing maternal mortality and morbidity. Use of the World Health Organization (WHO) Implementing Best Practices Knowledge Gateway, which requires only a low bandwidth connection, gave educators an opportunity to engage in the discussion even in the most Internet access-restricted places (e.g. Ethiopia). In order to sustain an online CoP, funds must come from an international organization (e.g. WHO regional office) or university that can program the costs long-term. Eventually, the long-term effectiveness and sustainability of GAPS rests on its transfer to the members themselves.

## Introduction

A community of practice (CoP) provides a means of gathering and sharing information. Popular in business, a CoP is an informal, self-selected group of people who share expertise and who are brought together to solve problems and share knowledge [1]. Evaluators of CoPs have noted that discussion within a CoP tends to be less constrained than discussions generated by more conventional methods, allowing for creative and novel solutions to old problems [1]. However, shared information within a CoP is frequently experiential, which may limit the validity of the evidence being shared [2].

The Capacity Project was a USAID-funded global initiative with multiple activities focused on strengthening human resources for health. The Project was led by IntraHealth International in collaboration with partners IMA World Health, Jhpiego, Liverpool Associates in Tropical Health (LATH), Management Sciences for Health (MSH), PATH and Training Resources Group, Inc. (TRG). In the pre-service education (PSE) arena, the Project has focused on strengthening key areas, such as family planning (FP) and HIV/AIDS, especially to address issues of poorly developed clinical competencies. This has included facilitating systems for developing and implementing competency-based curricula and harmonization of FP and HIV/AIDS content for pre-service and in-service training, especially of nurses and midwives [3].

The Capacity Project established the Global Alliance for Pre-Service Education (GAPS) project to provide a forum for the discussion of issues related to teaching and acquiring competence in FP. GAPS functioned as an electronic community of practice (CoP) housed within the World Health Organization (WHO)/Implementing Best Practices (IBP) Knowledge Gateway. The moderators of GAPS were inspired by the success of the GANM. The GANM CoP, moderated by the Johns Hopkins School of Nursing and hosted by the IBP Knowledge Gateway, exemplified the potential of this medium. Lathlean et al. [4] commented that CoPs provide the opportunity to reach practitioners and educators who traditionally might not have professional access to one another.

The GAPS CoP facilitated a virtual collaboration among educators from around the world to share relevant issues and explore common challenges associated with identifying and teaching FP core competencies. This method of sharing and eliciting information was based on the growing interest to understand how new information and communication technology may be used to support efforts to scale up and improve PSE in low-income countries [5].

GAPS was intended to build a community of stakeholders in PSE. The intended goal of the group of PSE stakeholders was to discuss how competencies in FP were locally defined and taught and eventually identify and share best practices and strategies. The leaders of GAPS hoped that this discussion would provide a critical understanding leading to a globally acceptable set of FP PSE core competencies.

This case study describes the process and outcome of GAPS and discusses the major issues that the CoP identified in teaching and learning FP competencies in low-resource settings.

### Defining competence

Competence can be defined as an "ability to do something well, measured against a standard, especially ability acquired through experience or training" [6]. This ability translates into performance and may be measured if standards are clear and well-established.

Competency as a health care provider requires knowledge acquisition in the classroom, practice in the skills lab and application of knowledge, skills and professional behaviour in the clinical practice setting. Producing competent health providers requires a competency-based curriculum and competency-focused assessment techniques.

The curricula of health worker education programs are often knowledge-focused and rely on resources that are out of sync with current evidence. Education programs tend to include material (based on Western medical text books and curricula) that is not directly applicable or relevant to prevalent health concerns in developing countries. As a result, curricula are long and may fail to address the key health issues [5]. Programs also lack competency-based clinical skills labs and often rely on clinical supervision by overburdened clinicians working in tertiary hospitals. These factors result in insufficient emphasis on competencies needed at the primary health care level [7].

## Case description

### The Global Alliance for Pre-Service Education (GAPS)

**Figure 1:**
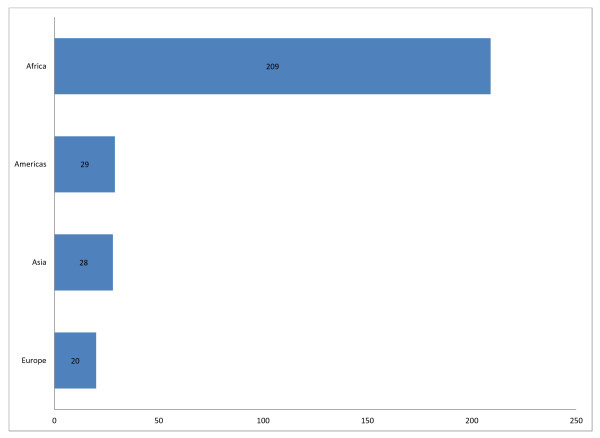
**GAPS Membership by Region**. GAPS drew 273 individual members, representing 49 countries.

GAPS drew 273 individual members, representing 49 countries worldwide. Approximately 65% of its members are living and working in low-resource settings in Africa, Asia and Central America. The remainder is comprised of members of universities and cooperating agencies in the United States, Canada and Europe (see Figure 1).

The moderators of GAPS ran three online forums, all of which attracted substantive membership and hosted dynamic discussions. The three discussion forums were:

1. A general discussion of FP competencies and competency-based training principles, which ran from January 16-February 16, 2008

2. A structured group analysis of existing FP competencies, which ran from March 3-14, 2008

3. An exchange of challenges and best practices associated with teaching the priority FP competencies, which ran from March 31-April 16, 2008.

Each forum had goals and objectives to guide the moderators. Questions that assisted in meeting the objective of each forum were posted online to the CoP. Following completion of each forum discussion, transcripts were distributed to a small group of experts in international FP and PSE for analysis; findings were collated and shared with the GAPS community with a request for further local insights.

## Discussion and evaluation

Each forum was analyzed by a group of experts in FP and PSE. Experts were asked to identify:

• Common themes from the discussion

• Challenges that were discussed

• Challenges that appeared to be specific to a country or a region

• Key strategies that were highlighted

• Relationship of the discussion to the forum objective.

### Forum one

#### Goal

The goal of Forum 1 was to explore the application of Competency-Based Education (CBE) principles to PSE of health care providers in low-resource settings. An emphasis was placed on the specific exploration of FP competencies.

#### Common themes

Common themes resulting from this forum were:

• There was a strong consensus on the relationship among competencies, CBE and the essential linkage to job-related performance standards.

• Most contributors defined competency as essential knowledge, skills and attitudes. Some added the concepts of clinical reasoning, knowing how to act and react to situations and solving complex problems, efficiency, confidence and the ability to mobilize resources.

• Competencies help delineate between roles in clinical practice which may prevent conflict of interest between different roles and levels of practice.

• Competencies should be used to guide the development of curricula and allocation of scarce academic resources.

• The assessment of student progress and readiness for practice should be based on competencies. Some examples of the use of Observed Structured Clinical Examinations (OSCE) were identified.

• Competencies must be demonstrable and measurable.

• It is important to ensure those responsible for curriculum development are competent in the subject matter.

• The effectiveness of CBE is enhanced by follow-up and mentoring.

• There is often poor linkage between national FP standards and competencies in the curriculum.

• No PSE core competencies were identified.

• GAPS members were reluctant to discuss specific FP core competencies.

#### Challenges

Challenges to CBE were identified as:

• Integration of specific content areas into the larger curriculum

• Non-measurable learning objectives

• Increasing student population without a corresponding increase in resources leading to shortcuts in curriculum development.

#### Strategy

The key strategy that was extracted was: competencies should be the basis for all curriculum development and implementation.

#### Relation to the objective

Relation to the objective was well-addressed by the question, as educators shared their definitions and understanding of 'competency' and described knowledge, skills, attitudes and abilities as integral to CBE.

### Forum two

#### Goal

The goal of Forum 2 was to have an analysis of competencies related to the provision of FP services by individuals deployed from health related training institutions in low-resource settings.

#### Common themes

Common themes resulting from this forum were:

• Competencies need to include non-clinical competencies such as those dealing with logistics, supply management, quality of care and leadership.

• Integration across subjects and across years of study must be reflected in the services as well as in the curriculum.

• Integration and strengthening of a broader curriculum will receive greater stakeholder buy-in.

• Attitude formation during learning is poorly covered.

#### Challenges

Challenges in competencies related to provision of FP services were not region-specific and included:

• Teaching and measuring the acquisition of 'attitudes' as compared to more concrete knowledge and skills.

• Teaching broader competencies that extend beyond tasks.

• FP is viewed as a minor topic.

• Feedback from the workplace to the classroom is missing and therefore preparation of graduates is incongruent with the needs of the workplace.

• Motivated and interested clinicians are needed to work with students.

• Instructors and staff lack the competencies required to assess and analyze competencies.

#### Key strategies

Key strategies included:

• Creating teams of students, enhancing appreciation of roles and team work in the workplace.

• Borrowing from the field of marketing to create awareness, attention, interest, desire, conviction and then action. Analyzing results from social marketing inquiries and focusing on what women want.

• Teaching attitudes by integrating this domain into the pre-service curriculum since attitudes take longer to develop than in-service training would allow for:

➢ Creating situations that allow for reflection and debate

➢ Clinical attachments and 'role-modelling'

➢ Community rotations that encourage community focus and understanding.

#### Implications

This forum suggests that FP competencies have not been sufficiently integrated into the curriculum in enough countries to merit an in-depth analysis. There are overriding issues that need to be addressed prior to addressing method-specific competencies. FP is still considered a minor topic and may often be omitted if the faculty member is not comfortable teaching the content.

### Forum three

#### Goal

The goal of Forum 3 was to analyze challenges and best practices associated with CBE aimed at the provision of FP services by graduates deployed from health related training institutions.

#### Common themes

Common themes resulting from this forum were:

• Majority of discussion was around HIV/AIDS, which revealed where much emphasis in programming is focused.

• There is a disconnect between theory and practice.

• Many instructors are not providing clinical services.

• The attitude of the instructor towards FP is important. If the instructor is not conversant in or is biased against FP, the mindset of the students may be affected.

Current resources and approaches are inadequate to prepare competent service providers.

#### Challenges

Some challenges were region-specific, particularly cultural and religious ones, but otherwise the challenges were universal. Predominately Catholic countries reported issues around contraception, and Muslim regions exhibited 'shyness' to discuss matters of sexuality and contraception. A number of challenges were repeated and also similar to the common themes:

• Deficiencies exist in the clinical practice area (e.g. site preparation and supportive learning environment).

• Cultural and social norms limit FP practice/participation among clients, faculty and students.

• There is a disconnect between the classroom and clinical practice.

• Students suffer from a lack of clinical opportunity to practice what they have learned in theory.

• There was an inability to locate target competencies in job-related documents.

• Issues exist of funding, coordinating and managing CBE to prepare competent providers.

• There is a lack of awareness if standards or job descriptions exist.

• There exists a lack of instructors and an ever-rising student-to-instructor ratio.

• There is an issue of contraceptive availability.

#### Key strategies

Key strategies for meeting some of these challenges included:

• Certification of health care workers.

• Post-basic or pre-deployment course on FP.

• Interventions raising awareness of faculty attitudes.

• Mandate to cover topics regardless of religious or cultural beliefs.

• Reducing the theory-practice gap with more simulated and real clinical practice.

• Preparing instructors in the development and delivery of competency-based strategies.

• Preparing instructors to assess student competencies.

• Strengthening clinical sites.

• Considering job-based training and e-learning to increase skills of clinical preceptors.

• Preparing students to evaluate their learning environment and provide feedback.

• Interventions should be on a national scale.

• Integration to get larger buy-in of stakeholders.

More challenges than best practices were identified. The literature suggests the importance of clear standards and core competencies that are clearly linked to accurate job descriptions. The key strategies identified in the forum lacked real strategic direction, which may demonstrate that participants, although interested to share, may have lacked the clear operational framework necessary for scaling up CBE.

#### Cost implications

The direct cost of GAPS was approximately US$ 21k over approximately eight months. Cost of similar CoPs may vary and depend on the cost of the moderators and indirect costs. However, an evaluation on feasibility and cost effectiveness was not done as the potential for this CoP to continue relies on further funding. The IBP Knowledge Gateway agreed to continue hosting the GAPS forum indefinitely.

## Conclusion

GAPS provided an important glance at the challenges and opportunities facing educators charged with preparing a health care provider workforce in the developing world. This robust conversation around the issues of CBE led to several important insights with practical implications for strategies aimed at PSE.

### Lessons learned

#### Implications for online CoP

There were several lessons learned in the process of running this online forum. Despite the activity and high membership, there were many silent members. Twenty-nine, or 16%, of the registered GAPS members contributed to ten active discussions. While this number of active contributors appears to be small, this percentage is favourable given the typical 10% ratio of active contributors to members reported on other IBP communities [8]. Additionally, had GAPS forums continued, we might hypothesize that the momentum would have led to increased membership and greater direct participation based on the trend occurring in GAPS, as well as observations seen in the GANM CoP. While we understand that online CoPs do not engage everyone, they provide an important opportunity to engage the larger community.

The moderator ensured full exploration of each forum topic. There were times, however, where educators expressed a desire to share issues tangential or unrelated to the forum topics. On certain occasions, when members wanted to express ideas or share information unrelated to the forum topic, they were provided with an alternative space within GAPS for this purpose.

The Knowledge Gateway provided an excellent means of reaching out to a broad interdisciplinary array of educators as well as NGOs actively engaged in support of PSE in low-resource settings. Members of the community were anxious to connect with one another and offered their appraisal of the challenges that they faced in their environments. While the conversation may have been somewhat skewed by differing quality of and access to computers and the internet, the themes that emerged from analysis of the varied points of view of the members was noted.

**Figure 2:**
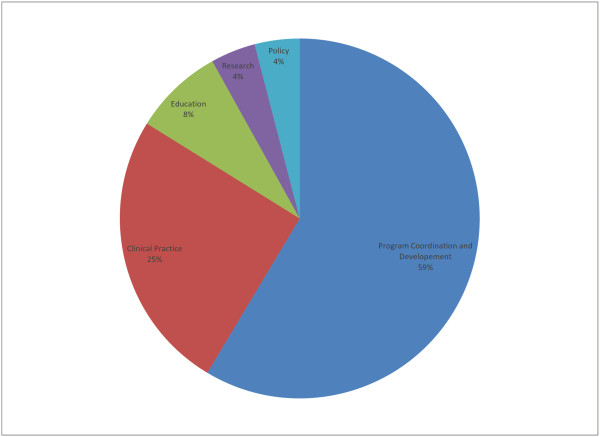
**GAPS Membership by Cadre**. The interdisciplinary GAPS membership allowed for a comparison and contrast of competencies needed by different members of the health care team in order to effectively deliver FP services.

In service delivery areas where cadres had distinct roles in FP management, the interdisciplinary community provides an opportunity to discuss important collaborative linkages (see Figure 2). In addition, promising practices, documents and other knowledge-sharing may occur in an online format.

CoPs require external support while in development in order to succeed. GAPS membership in its early stages was skewed toward members of international nongovernmental organizations with an interest in PSE but eventually became more populated with grassroots educators working in the targeted low-resource settings. If external funds from stakeholders of pre-service are utilized, these funds must be from an international body (e.g. WHO regional office) or university that can program the costs long-term. However, eventually, the long-term effectiveness and sustainability of GAPS rests on its transfer to the members themselves, who must be encouraged and mentored in order to take on this role.

#### Implications for promotion of CBE

Dissemination of a consensus definition of competency is fundamental to any efforts aimed at preparing effective health care providers. Target competencies must be logically linked to standards that have been adopted by the national health care systems, analyzed against realistic expectations of new graduates entering the workforce and fully vetted by both the clinical and the academic communities prior to their inclusion in the curriculum. In addition, resources aimed at competency development must be appropriate for local delivery of services and not based on tertiary-level Western medical practices. While Western texts and curricula may be useful for their technical information, they should be used strategically as they do not represent all the public health needs or resource limitations.

While competencies must be specified in the job description of each cadre of health provider, their development and application have several cross-disciplinary implications. The interdisciplinary GAPS membership allowed for a comparison and contrast of competencies needed by different members of the health care team in order to effectively deliver FP services. For example, in some instances where tasks have been shifted from physicians to nurses, identical competencies are needed in both the medical and nursing curricula, especially considering that physicians would be expected to train nurses. In these cases, discussion within an interdisciplinary community can result in shared opportunities, lessons learned and teaching strategies.

The developmental status of students, allocation of scarce clinical and academic resources, space within an already crowded program of study and clinical competency of available faculty must all be considered carefully as part of the decision-making when integrating FP clinical competencies within a curriculum. Interestingly, GAPS members have suggested a focus on several professional competencies (e.g. communication, leadership, cultural sensitivity, teamwork and problem solving) that would enhance the resulting health care graduate to operate in a complex health environment. Participants suggested the inclusion of these professional competencies would provide a strong foundation for acquiring other competencies needed in the workforce beyond the clinical domain.

### Recommendations

#### Recommendations for Online CoPs

GAPS provided a forum for discussion of the opportunities and challenges that are associated with implementing a competency-based curriculum, with an attempt to discuss specific FP competencies. Due to funding limitations, GAPS was unable to have a face-to-face meeting to engage the most active participants from various parts of the world. While the GAPS CoP was solely internet-based, CoPs are most effective when there are venues for colleagues to gather together, discuss, share best practices and learn strategies from one another. The GAPS leaders found that these opportunities do exist at global conferences. Participating in global conferences and sharing results contributes to raising awareness of the needed strategies to strengthen PSE, network building, and improved training that will increase the number of competent providers in FP and clinical preventative care.

#### Recommendations for promotion of CBE

Currently, health care curricula focus primarily on knowledge acquisition and then on psychomotor skills development. Given the complexities of emerging health care systems and the great disease burden facing health care providers, inclusion of clinical decision-making capacity within the definition of competency is critical. Increased attention directed toward educational strategies such as problem-based learning and use of role-plays, simulations and structured clinical mentoring will enhance development of clinical decision-making.

Resources to support CBE in the academic setting must be sufficient and appropriately distributed. Faculty and students must have access to evidence-based literature. Skills labs containing clinical equipment and supplies that match service delivery standards must be in place. Organizing lab stations around each of the target competencies will have positive learning and assessment implications.

Improved linkages between educational institutions and health care facilities are also essential to the development of target competencies. Preceptors responsible for teaching students in the clinical setting must be actively involved in developing teaching strategies and assessment tools used both in the skills labs and clinical settings. Discordant expectations are a major source of frustration to students, instructors, and preceptors and cause significant interference with learning. Clear objectives assist both the faculty and the students to realize their expectations of each other with the resources that are available.

Where clinical competencies are incorporated into PSE, responsible instructors and preceptors must be clinically proficient. Faculty and preceptors must also be prepared to teach to and assess the target competencies in the classroom, skills labs and clinical settings. These essential prerequisites may require a significant investment in training and institutional strengthening prior to integration of new clinical competencies into a curriculum. To maximize success of this complex, long-term PSE strengthening process, a broad array of academic, clinical and governmental stakeholders should be consulted throughout.

## List of abbreviations

CBE: Competency-Based Education; CoP: Community of Practice; FP: Family Planning; GANM: Global Alliance for Nursing and Midwifery; GAPS: Global Alliance for Pre-Service Education; IBP: Implementing Best Practices Knowledge Gateway; LATH: Liverpool Associates in Tropical Health; MSH: Management Sciences for Health; NGO: Non-governmental Organization; OSCE: Observed Structured Clinical Examinations; TRG: PATH and Training Resources Group; PSE: Pre-service Education; USAID: United State Agency for International Development; WHO: World Health Organization

## Competing interests

The authors declare that they have no competing interests.

## Authors' contributions

AUT assisted with the concept of the GAPS community of practice, the implementation of the forums, the financial oversight of the project, the acceptance of submissions to the online community of practice, the organization of the online resources for the community of practice, writing and submission of the project report and creation of analysis framework for the analysis team. AUT also is responsible for the concept of the paper to share results and lessons learned, as well as literature review, writing and submission of this paper's outline, abstract and content.

GPF assisted with the implementation of the forums, literature review, writing content for the paper and the creation of the diagrams and legends.

PJ assisted with the concept of the GAPS community of practice, the framework for implementation, the moderation of the forums, analysis of the forums, and he contributed to the writing of the project report and writing content for the paper.

BS assisted with the concept of the GAPS community of practice, the analysis of the forums, and she contributed to the writing of the project report, literature review and writing content for the paper.

All authors read and approved the final manuscript.

## Authors' information

AUT is a Senior Technical Advisor, Global Learning Office at Jhpiego. She is a public health specialist and registered nurse with experience in family planning, pre-service, emergency nursing, and breastfeeding. She also holds an adjunct faculty member position at the Johns Hopkins University School of Nursing. AUT provides technical assistance globally to Jhpiego's country programs in family planning and pre-service. She has particular expertise in clinical training approaches, competency-based training, malaria, counseling in family planning methods and HIV counseling and testing and developing job aids and resources for providers and faculty. AUT also volunteers at Planned Parenthood Association of Maryland as a family planning and HIV counselor and clinician.

GPF is a first year MD/MPH student at Thomas Jefferson University and received a BA in Public Health from Johns Hopkins University. She is also an active volunteer with Planned Parenthood.

PJ is Director of the Global Learning Office at Jhpiego. He is a nurse-midwife and educational psychologist with nearly 20 years of experience as a pre-service educator and program administration. PJ has expertise in instructional design, measurement of learning outcomes, academic program accreditation, educational needs assessment, application of learning technologies and certification and licensure of health providers. He currently provides global technical assistance in areas related to the education and training of health care providers.

BJS is Director of Technical Leadership at IntraHealth International and at the time of the GAPS case study reported here, she was a Senior Advisor for the Capacity Project. BS is a health workforce development specialist, with 25 years of experience in improving workforce performance.
